# Loss, persistence and reversal of phenotypic traits

**DOI:** 10.1002/brv.70168

**Published:** 2026-03-30

**Authors:** Giobbe Forni, Giuseppe Fusco, Filippo Nicolini, Heather Bruce, Andrea Luchetti

**Affiliations:** ^1^ Department of Biological, Geological and Environmental Sciences University of Bologna via F. Selmi 3 Bologna 40126 Italy; ^2^ Department of Biology University of Padova via U. Bassi 58/b Padova 35131 Italy; ^3^ Division of Genetics and Genome Biology School of Biological and Biomedical Sciences, University of Leicester University Rd. Leicester LE1 7HR UK; ^4^ Department of Zoology University of British Columbia #3051 – 6270 University Blvd Vancouver BC V6T 1Z4 Canada

**Keywords:** Dollo's law, pleiotropy, plasticity, hemiplasy, gene regulatory network

## Abstract

The irreversibility of complex trait loss has long been a tenet of evolutionary biology. However, this idea is increasingly at odds with the numerous documented exceptions across the Tree of Life. We synthesise this growing body of evidence across a diverse array of taxa and traits, exploring the evolutionary conditions that enable evolutionary reversal. By integrating macroevolutionary, genetic, and developmental information, we argue that trait reversal is commonly fostered by some form of persistence in the generative developmental pathway of the lost trait. We identify three overarching modes of trait reversal and support them with multiple case studies: by pleiotropy (the involvement of the same generative components in other traits and/or functions), by plasticity (environment‐dependent expression of the trait) and by hemiplasy (persistence in another lineage, followed by reticulate evolution). We also examine important affinities between trait reversal and evolutionary novelties, undermining a neat distinction between what is old and what is new in evolution. This survey may provide a useful framework for future explorations of the developmental mechanisms underlying these still overlooked macroevolutionary dynamics.

## I. INTRODUCTION

Evolutionary change does not always take the appearance of a modification of pre‐existing features. Sometimes a change can emerge in the form of a disruptive *novelty*, something that has puzzled evolutionary biologists since the birth of the discipline (Wagner & Lynch, 2010). Still, evolution can exhibit a different, but equally striking pattern of change: the reinstatement of a lost trait, or *trait reversal*. Although instances of this phenomenon have been widely documented across the Tree of Life, trait reversal has received little attention compared to evolutionary novelties. While in the latter the unexpectedness stems from the innovative nature of the newly emerged feature, in trait reversal what is surprising is the restoration of an old feature that was believed impossible to recover and thus lost forever.

Evolutionary change may be reversible or irreversible depending on various factors, in particular the amount of time elapsed since the change and its magnitude (Bull & Charnov, 1985; Teotónio & Rose, 2000; Porter & Crandall, 2003; Powell, Price & Kronauer, 2020; Pla *et al*., 2022). However, it has long been argued that the reappearance of a complex trait after its loss, in exactly its ancestral form, is highly improbable, as it would require evolution to retrace the steps necessary to evolve the trait in the first place. This idea is commonly referred to as Dollo's law (Dollo, 1888; Gould, 1970), which in its original formulation states that *an organism cannot return, even partially, to a former state already realised in the series of its ancestors*. Notably, this formulation does not preclude the re‐emergence of a trait, as long as this does not fully replicate its ancestral condition. However, contemporary interpretations of Dollo's law tend to treat any reappearance of a lost trait as a violation of the law, mostly in disregard of the degree of similarity between the ancestral and the regained traits. Apparent violations of Dollo's law, under this modern interpretation, are frequently reported across the Tree of Life (e.g. Collin & Miglietta, 2008; Elmer & Clobert, 2024).

By integrating macroevolutionary, genetic, and developmental information, we review the evidence for the apparent reinstatement of lost traits, exploring the evolutionary conditions that enable trait reversal. We argue that trait reversal is commonly fostered by some form of persistence in the generative developmental pathway of the lost trait, of which we provide a process‐based classification. We also examine important analogies between trait reversal and evolutionary novelties.

In advance of our argument, a few terminological notes are in order. We have adopted an unloaded concept of ‘trait’ (or ‘character’), which we decided to define as any feature of an organism that can be described or measured (Mayr, Linsley & Usinger, 1953). This is a standard use of the term in comparative biology and phylogenetics. Under such a broad conception, a trait can be anything from an anatomical body part (which can be present or absent) to an attribute (whether molecular, morphological, anatomical, or physiological) that applies to the entire organism or to a specific body part (Fusco & Minelli, 2023), contingent on the possibility of quantification or qualitative description. Life cycles and life‐history traits are also included. Characters have ‘states’, which are values or conditions dependent on the form of description adopted. The expressions ‘trait loss’ and ‘trait reversal’ are shorthand for specific patterns in character state transitions. The first term refers to the disappearance of a specific character state, while the latter refers to a double transition from character state A to state B and back to A. As a macroevolutionary pattern, it is the restoration of traits of a certain degree of complexity that poses particular challenges and requires special explanations, since simple (or simply controlled) traits can alternate between states quite easily (e.g. Kratochwil *et al*., 2018). In quantitative genetics, a ‘complex trait’ is defined as one controlled by multiple genes and influenced by various environmental factors. For our purposes, we have adopted a broader, phenotype‐based definition of complex trait, that allows inclusion of cases where the genetic control of a feature is unknown, but its phenotypic characteristics clearly indicate the involvement of multiple underlying developmental factors.

## II. TRAIT REVERSALS ACROSS THE TREE OF LIFE

**Fig. 1:**
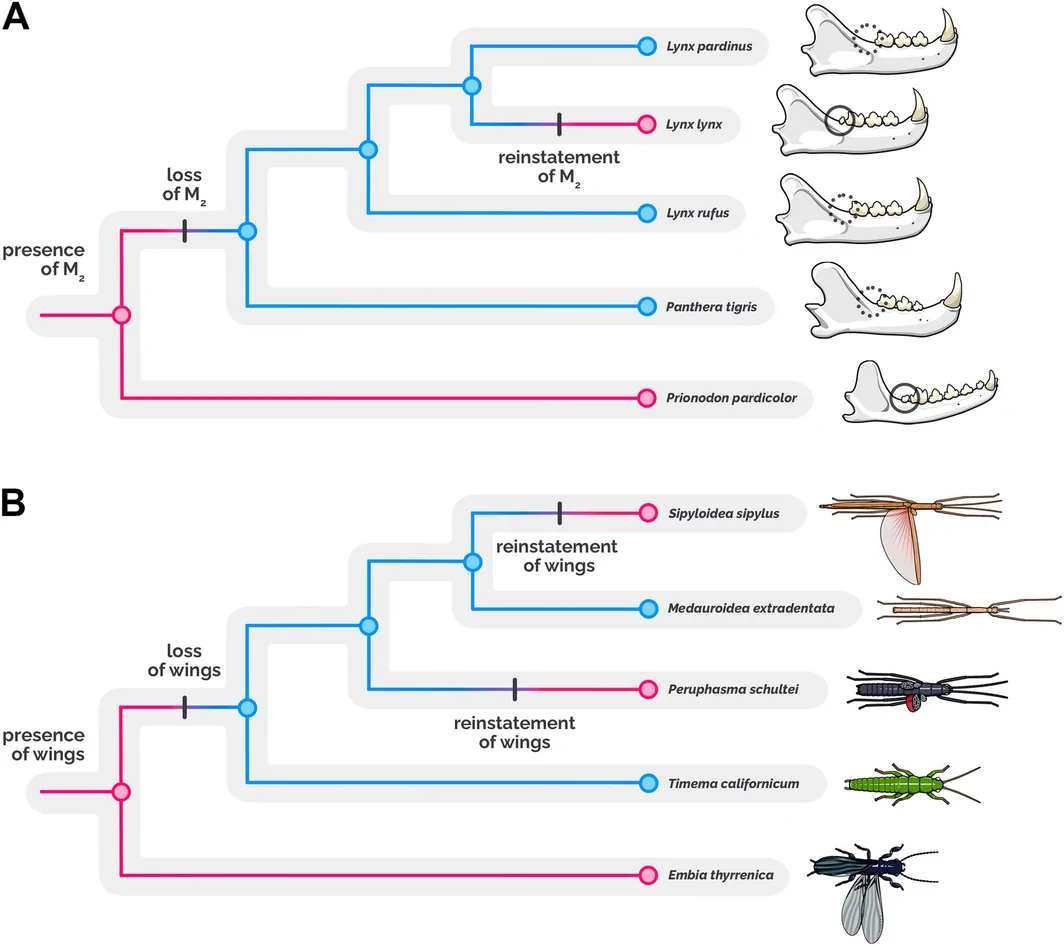
Examples of trait reversals. (A) In several extant populations of the Eurasian lynx (*Lynx lynx*), the second lower molar (M2) has been reacquired, after its loss in the common ancestor of felids (adapted from Lynch, 2023). (B) In different lineages of stick and leaf insects (Phasmatodea), the wings have been reacquired after this feature was lost in the last common ancestor of the taxon (adapted from Forni *et al*., 2022). Pink = nodes and branches exhibiting the ancestral state of the character; blue = nodes and branches exhibiting the derived state of the character; dashes highlight loss and reinstatements. Phylogenetic trees are cladograms, and their branch lengths are not to scale.

Most evidence of complex trait reversals (also referred to as reversible evolution, re‐evolution, regain, reinstatement; Elmer & Clobert, 2024) comes from character state reconstruction along the branches of phylogenetic trees, using comparative phylogenetic methods. A classic case of reversal, among those that have been proposed, is the reinstatement of the second lower molar in the Eurasian lynx (*Lynx lynx*), a tooth that is absent in all other felids, including other extant and extinct lynx species (Fig. 1A; Werdelin, 1987; Lynch, 2023). The second lower molar is thought to have primitively been lost in the lineage leading to Felidae, and to be subsequently reacquired only in this species. Stick and leaf insects (Phasmatodea) offer another clear example of reversal. Although many extant lineages are winged, phylogenetic evidence suggests that their last common ancestor was wingless, implying that wings have re‐evolved independently multiple times in the group (Fig. 1B; Whiting, Bradler & Maxwell, 2003; Bank & Bradler, 2022; Forni *et al*., 2022; Yang *et al*., 2023).

As in the case of the lynx, some instances of reversal concern the reappearance of lost elements in serially homologous series, such as mandibular teeth in frogs (Wiens, 2011; Paluh *et al*., 2021), and digits in *Bachia* and *Brachymeles* lizards (Kohlsdorf & Wagner, 2006; Siler & Brown, 2011). Other examples are offered by the reappearance of highly complex structures that are not part of a serially homologous series, such as the eye in ostracod crustaceans. Syme & Oakley (2012) observed that eye presence in different species is correlated with habitat depth, from which they inferred reversal to compound eyes from eyeless ancestors associated with multiple transitions from deep to shallow waters. Similarly, trait reversal from degenerate to functional eyes has been suggested for cave fishes (genera *Astyanax* and *Sinocyclocheilus*) living near karst windows, where natural collapses in cave roofs allow light to penetrate (Espinasa & Borowsky, 2000; Mao *et al*., 2021). Other notable examples include metamorphosis in ambystomatid salamanders (Shaffer, 1984), the coiled shell in the gastropod *Trochita trochiformis* (formerly *T. calyptraeformi*s; Collin & Cipriani, 2003), the presence of limbs in several squamate species (Brandley, Huelsenbeck & Wiens, 2008; Skinner, Lee & Hutchinson, 2008), sexual ornaments in birds and insects (Kraaijeveld, 2014), the shape of sex combs on the legs in several species of *Drosophila* (Seher *et al*., 2012), flower pigmentation in different *Petunia* species (Esfeld *et al*., 2018), ancestral eggplant‐type alkaloid isomers in wild Solanaceae from the Galápagos (Jozwiak *et al*., 2025), surface‐dwelling phenotype in a cave salamander (*Proteus anguinus*; Recknagel *et al*., 2023), and skeletal elements in chameleons (Diaz & Trainor, 2015). Examples of morphological features that were lost and subsequently reinstated are currently so numerous to make it almost impossible to compile a comprehensive list.

Besides morphological structures, reversible evolution has also been claimed for life‐cycle and life‐history traits. The reappearance of a larval stage after its loss was inferred for *Bembicium* gastropods (Cummins *et al*., 2022) and several amphibians (Liedtke, Wiens & Gomez‐Mestre, 2022), including frogs (Wiens *et al*., 2007), salamanders (Chippindale *et al*., 2004) and caecilians (San Mauro *et al*., 2014). Similarly, different crustacean groups have lost and regained their ancestral nauplius stage, which is the earliest larval stage in the crustacean life cycle (Dahms, 2000). Other notable examples include self‐incompatibility in the daisy family (Asteraceae; Ferrer & Good‐Avila, 2007), oviparity in lizards and snakes (Squamata; Lynch & Wagner, 2010; Recknagel, Kamenos & Elmer, 2018), a free‐living lifestyle in parasitic protists and mites (Cruickshank & Paterson, 2006; Klimov & O'Connor, 2013; Xu *et al*., 2016) and placentotrophy in cyprinodontiform fishes (Helmstetter *et al*., 2016). Trait reversal has been argued also for a key feature of the eukaryote body plan: the return to unicellularity in yeast populations that recently evolved multicellularity (Rebolleda‐Gómez & Travisano, 2019) and, conversely, the restoration of multicellularity in some lineages of Zygnematophyceae algae (the sister taxon of land plants), after the last common ancestor of the group regressed to the unicellular condition from more complex ancestors (Hess *et al*., 2022).

Many potential pitfalls can challenge our understanding of trait evolution, especially in the context of phylogenetic comparative methods. For example, the inferred pattern can be influenced by (*i*) species sampling, (*ii*) the robustness of the underlying phylogenetic hypotheses, encompassing both the tree topology and branch lengths (Galis, Arntzen & Lande, 2010; Rangel *et al*., 2015), (*iii*) heterogeneity of rates of phenotypic evolution among the branches (Skinner, 2010), (*iv*) intraspecific variation and measurement errors of the trait (Paterno, Penone & Werner, 2018), and (*v*) the effect of the character state on rates of speciation and extinction (Sauquet *et al*., 2017; Bailey, Pascoal & Montealegre, 2019). In addition, specific methodological flaws in the use of phylogenetic comparative methods for testing reversible loss have been exposed (Goldberg & Igić, 2008), advocating for the integration of developmental and molecular approaches (Griffith *et al*., 2015). Although these methodological criticisms can question some individual claims of trait reversals, many others have proved to be robust to testing with the most recent analytical approaches. These include the reversal of biphasic life cycles in frogs (i.e. the larval stage; Liedtke *et al*., 2022), oviparity in Squamata (Esquerré *et al*., 2019) and wings of stick insects (Bank & Bradler, 2022; Forni *et al*., 2022, 2023).

All these examples apparently violate Dollo's law: trait reversal seems to be not so unfeasible, after all. Indeed, an ever‐growing body of evidence strongly supports the view that evolution can reinstate traits, at least in a form which is broadly similar to that once lost in the ancestors of extant species. However, most of these claims are grounded in the simple inference of a specific palindrome pattern of character state transition (A–B–A) in a phylogenetic tree, while the mechanisms through which the reversal may occur remain largely overlooked. Here is where modern developmental biology and molecular genetics can come to the rescue. By elucidating the molecular mechanisms involved in phenotype formation, numerous studies in this field suggest that the loss of a trait does not necessarily entail the loss of all the underlying generative components of the phenotype.

## III. PERSISTENCE OF (PHENOTYPICALLY) LOST TRAITS

Although ‘trait loss’ is a term that seems to imply a very neat definition of this evolutionary change, there is instead a broad spectrum of phenotypic conditions and developmental patterns that are observed. At one end of this spectrum are traits that have completely disappeared and are absent throughout development, showing no morphological signatures of their ancestral presence, like limbs in Colubroidea snakes (Cohn & Tickle, 1999). At the other end are traits that begin to develop but are highly reduced or completely suppressed in later developmental stages (Sadier, Sears & Womack, 2022). These traits are often referred to as *vestigial*, when they are still present in a rudimentary form but have lost their original function (Müller, 2002), or *atavistic*, when they are occasionally reinstated only in some abnormal individuals (Hall, 1984). The phenotypic loss of traits in adult stages often does not result from a failure to initiate their development, but rather from the subsequent degeneration of the trait, either through the loss of cell maintenance signals or from the induction of programmed cell death (Bejder & Hall, 2002; Leal & Cohn, 2018). This may reflect the highly interconnected nature of the patterning genes that initialise the development of an organ or a structure, such that it is easier to degenerate it rather than prevent its formation entirely.

Even when there are no remaining visible developmental traces of a lost trait, elements of the gene regulatory network (GRN) once involved in its development can survive the phenotypic loss. Complex phenotype development is regulated by the expression of large numbers of genes, which can undertake divergent evolutionary trajectories after the trait expression is lost. This partial persistence is exemplified in the visual system of cavefishes: in species from two non‐sister genera (*Sinocyclocheilus* and *Lucifuga*), only a subset of vision‐related genes has degenerated into pseudogenes, while others have remained intact in their sequence (Policarpo *et al*., 2021). A limited amount of pseudogenisation in genes related to traits that are lost in the evolution subterranean lifestyle (like vision and pigmentation) has been observed also in cave populations of the Mexican cavefish *Astyanax mexicanus*, which has been explained by both the relatively recent colonisation of the subterranean environment and the pleiotropic function of some of these genes (O'Gorman *et al*., 2021; Culver, Kowalko & Pipan, 2023; see Section IV). Another example is the glucuronic acid pathway in primates, including humans, which have lost the ability to synthesise vitamin C: while the terminal enzyme L‐gluconolactone oxidase has been disrupted by a frameshift mutation, the genes that form the rest of the pathway have remained intact, due to their essential roles in other fundamental metabolic processes (Lachapelle & Drouin, 2011).

The decay of the GRN underlying the development of a trait does not necessarily occur immediately, and it is not always complete, both in terms of the elements of the network and the structure of their interactions. While signs of GRN degradation, such as mutations in coding or regulatory regions (Langille *et al*., 2022; Sackton *et al*., 2019; Roscito *et al*., 2022) or complete gene losses (Yang *et al*., 2016), are common, many cases also show surprising persistence of these networks.

The preservation of the GRN that patterned lost elements of a series of repetitive structures, such as the reappeared second molar of *Lynx lynx* (Fig. 1A), is somewhat expected, since the elements of serially homologous groups share a large part of their developmental pathways (Hall, 1995; Kohlsdorf, 2023; Fusco, 2022). However, GRN persistence is not limited to serial homologs. Functional visual opsins are expressed in distantly related crustacean species of the families Amphipoda and Decapoda that independently colonised cave habitats and have highly reduced or absent eyes (Carlini, Satish & Fong, 2013; Stern & Crandall, 2018). In the parasitic plant genus *Cuscuta*, genes involved in photosynthesis are found to undergo strong purifying selection, even though these plants obtain the products of photosynthesis from their host plants (McNeal *et al*., 2007). In annelids that have independently lost the ability to regenerate heads, latent regenerative capacities can still be elicited, suggesting that much of the underlying regeneration machinery remains intact (Bely & Sikes, 2010).

GRN persistence can be elusive, such as in the case of the loss of teeth in the lineage leading to modern birds, which never reverted in any known extant or extinct species (Louchart & Viriot, 2011; Brocklehurst & Field, 2021). Yet, the early signalling pathway underlying tooth formation in mammals and non‐avian reptiles – presumably ancestral to Amniota – has been largely retained in birds (Kollar & Fisher, 1980; Chen *et al*., 2000; Mitsiadis *et al*., 2003). Remarkably, tooth development in chickens can be artificially induced by reactivating a program resembling that observed in the first‐generation teeth of alligators (Harris *et al*., 2006). However, these teeth do not form enamel or dentine, and the genes involved in the synthesis of enamel and dentine appear to have degenerated in birds (Sire, Delgado & Girondot, 2008). Shared upstream patterning in teeth, feathers, and scales (Dhouailly *et al*., 2019; Hulsey *et al*., 2020) may explain why feathers and scales in birds preserved enough of the ancestral GRN to permit tooth bud formation in experimental chickens.

Similar competence has been experimentally revealed in cypriniform fish, which develop functional teeth in the ventral posterior pharynx but, unlike other teleost fishes, do not possess oral teeth (Aigler *et al*., 2014). However, the inhibition of endogenous retinoic acid degradation is sufficient to restore oral tooth induction (Jackman *et al*., 2024). Artificial manipulations have been able to reinstate numerous lost traits that were never re‐established by natural evolution, such as the experimental induction of males in exclusively parthenogenetic cladocerans (Kim, Kotov & Taylor, 2006).

A major determinant of the fate of a trait (and of the underlying developmental and genetic pathway) following environmental shifts or changes in life history, is expected to be its relationship with the organism's fitness (Lahti *et al*., 2009). When the maintenance of the trait represents a fitness cost, natural selection is expected to lead to its adaptive decay and, eventually, loss. An excellent example of this phenomenon is the cavefish *A. mexicanus*, where genes underlying costly decayed traits (e.g. pigmentation, eyes, and circadian rhythms) show signatures of positive selection for pseudogenisation, a pattern not observed in surface fish populations (Moran *et al*., 2023). On the other hand, if a trait has no significant impact on the organism's fitness, the trait is expected to degenerate at a slower pace through the fixation of mutations that have a deleterious effect on the trait itself, but that are selectively neutral at the level of the whole organism. This seems to be the case for genes associated with male sexual traits following the transition to parthenogenesis. Since male‐specific traits are no longer expressed and subject to selection, they undergo gradual decay through the accumulation of neutral mutations (Kampfraath *et al*., 2020). By contrast, female‐specific traits, which are expressed in parthenogenetic females and thus exposed to the effect of selection, experience a non‐neutral and rapid degradation (Minelli & Fusco, 2006; van der Kooi & Schwander, 2014; Kraaijeveld *et al*., 2016).

Empirical and theoretical studies suggest that phenotypic trait loss does not necessarily entail the immediate or complete degradation of the underlying molecular machinery. For example, GRNs associated with the development of specific body structures (or the entire body plan) often present a deeply conserved ‘kernel’ (Davidson & Erwin, 2006), shared across lineages, such as that active during the ‘phylotypic period’ (reviewed in Cridge, Dearden & Brownfield, 2021). By contrast, evolutionary innovation and morphological diversity are likely to stem from changes in the more evolutionarily flexible downstream components of the network, i.e. those controlling morphogenesis and terminal differentiation. Numerous examples support this view, showing that while patterning and identity genes remain conserved, downstream terminal differentiation genes are often modified or lost. As discussed earlier, genes that define the initial odontogenic region in mammals are still expressed in toothless birds (e.g. *Pitx2* and *Pax9*; Chen *et al*., 2000), whereas downstream genes underlying enamel and dentine are non‐functional (*AMELX* and *AMBN*; Meredith *et al*., 2014). While certain genetic components may accumulate mutations or be lost, others can persist over immense evolutionary timescales, maintaining the potential for reactivation.

Trait reversals have been documented across remarkable evolutionary spans, including oviparity in a species of the sand boa genus *Eryx* [after ~60 million years (My); Lynch & Wagner, 2010], mandibular teeth in frogs (after ~200 My; Wiens, 2011), and the galactose (GAL) pathway cluster and galactose metabolism in yeast [after ~350 My (Shen *et al*., 2018; Haase *et al*., 2021)]. Computational simulations indicate that, while the probability of reactivating the protein coding sequence of a long coding sequence gene declines sharply after approximately one million years, smaller genomic elements, such as transcription factor binding sites or protein–protein interaction motifs (<100 bp), can remain functionally restorable for tens of millions of years (Marshall, Raff & Raff, 1994; Lynch, 2023).

The fact that in many cases, a trait seems to have been lost only at the phenotypic level, while the underlying GRN persists, suggests different mechanisms by which a trait can be reinstated. The next three sections examine some non‐mutually exclusive modes of trait reversal, classified on the basis of the underlying developmental or evolutionary mechanism of persistence: (*i*) pleiotropy, where elements of the developmental systems of the lost trait are preserved due to their roles in the development of other traits; (*ii*) phenotypic plasticity, where intermittent selective regimes sustain the preservation of an alternative phenotype over time; and (*iii*) hemiplasy, where key genes of the lost trait are maintained in closely related lineages, to be recovered at a later time through gene transmission that does not follow species phylogeny.

## IV. REVERSAL BY PLEIOTROPY

Pleiotropy refers to the dependence of multiple traits on the same gene or GRN, meaning that disruption of the shared gene(s) will affect every trait (Zhang, 2023). Thus, one possible mechanism of trait restoration after phenotypic loss is *via* the conservation of the trait's underlying genetic components due to the necessity of these components in the development of other traits in the same species.

Clear examples of trait reversal through pleiotropy are offered by traits that correspond to structural elements of a group with serial homology (Fusco, 2022), where the developmental program associated with the lost element remains active in other elements of the series (Jackman, Lynch & Gibert, 2025). Vertebrate teeth provide compelling examples, such as case of the second lower molar in lynxes presented above, where the developmental program required for its reinstatement was likely preserved by the other teeth (Lynch, 2023; Fig. 1A). Another example is the reappearance of mandibular teeth in *Gastrotheca* frogs, where the tooth GRN in the lower jaw was first deactivated and then reactivated while remaining continuously expressed in the upper jaw (Wiens, 2011; Paluh *et al*., 2021). Additional instances of serially repeated elements that can reversibly change in number are offered by the digits of fore‐ and hindlimbs in lizards of the genera *Brachymeles* (Wagner *et al*., 2018) and *Bachia* (Kohlsdorf, 2023; Kohlsdorf & Wagner, 2006).

However, pleiotropy can also facilitate the reversal of traits that are not unambiguously associated with serial homology. In ants, several examples illustrate this mechanism. In *Pheidole* ants (Myrmecinae), the super‐soldier subcaste appears to have been reestablished based on pleiotropic genes involved in the development of the standard soldier caste (Rajakumar *et al*., 2012). In Formicinae ants, ocelli have been reinstated independently in the worker caste on two separate lineages. Interestingly, in species closely related to those in which ocelli have been reinstated, the GRN responsible for ocellar development remains active throughout larval stages. This persistent expression likely reflects the multiple roles of GRN components in patterning the eye‐antennal imaginal disc of workers (Vasquez‐Correa *et al*., 2025). In another example within insects, pleiotropy has been proposed as an explanation for wing reversal in stick insects (Forni *et al*., 2022; Fig. 1B). This hypothesis is supported by the fact that insect wings share the same genes and developmental mechanisms with a diverse array of body wall outgrowths found throughout arthropods (Ohde, Yaginuma & Niimi 2013; Linz & Tomoyasu, 2018; Bruce & Patel, 2020, 2022; Clark‐Hachtel & Tomoyasu, 2020), including tergal plates, which are present in stick insects and thus available to preserve the wing GRN even in the absence of wings.

**Fig. 2:**
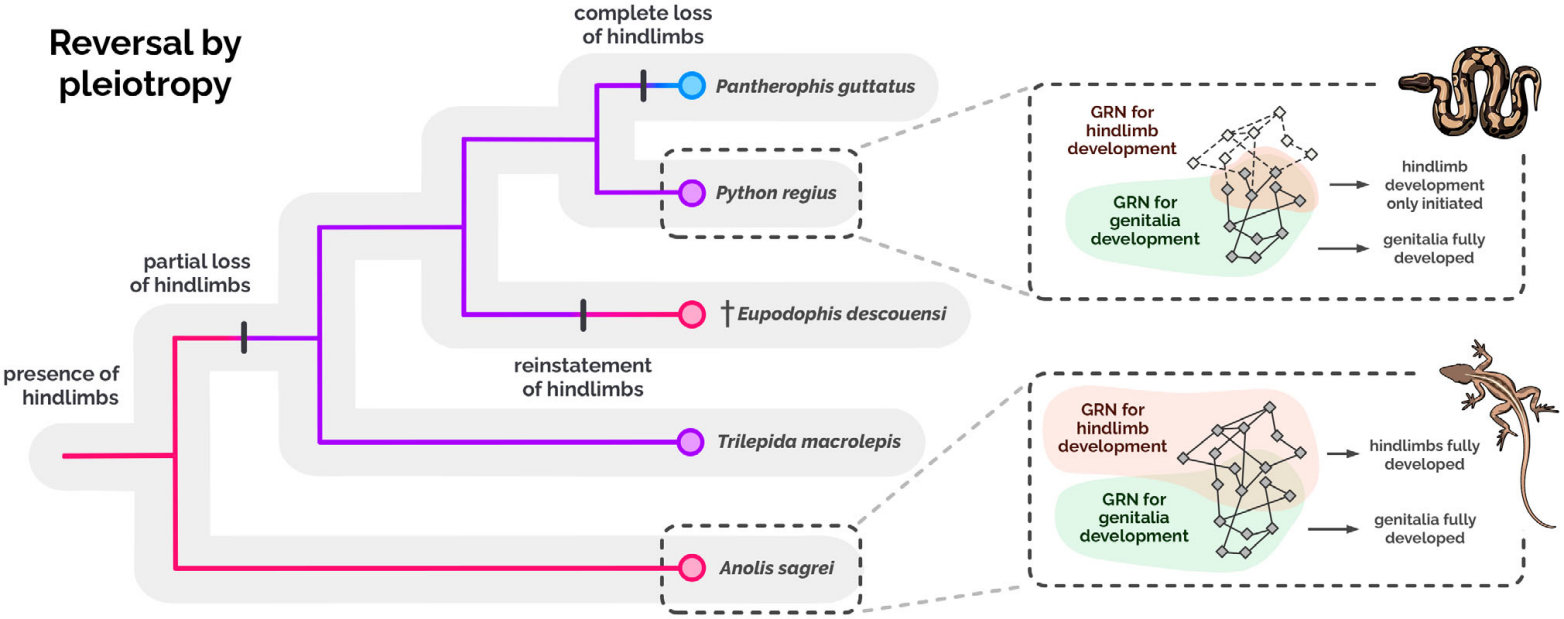
Reversal by pleiotropy. Pleiotropy—where a single gene influences multiple traits—can preserve developmental potential and facilitate the reappearance of lost traits. Following the evolutionary loss of hindlimbs in snakes, the gene regulatory network (GRN) underlying hindlimb development remains partially active in relatively basal snakes such as *Python regius*, due to its pleiotropic role in genitalia development. In extant species, this residual activity results in transient limb bud appearance in the embryo. In fossil lineages such as *Eupodophis descouensi*, fully developed hindlimbs reappeared, likely through reactivation of these shared GRN components. The cladogram topology is derived from Leal & Cohn (2016). Pink = nodes and branches exhibiting the ancestral state of the character; blue = nodes and branches exhibiting the derived state of the character; purple = nodes and branches exhibiting the intermediate state of the character; dashes highlight losses and reinstatement.

An emblematic example of putative trait reversal sustained by pleiotropy is found in several Cretaceous snake species, such as *Eupodophis descouensi*, *Pachyrhachis problematicus*, and *Haasiophis terrasanctus*, which exhibited well‐developed hindlimbs despite their placement within lineages that had previously lost functional limbs (Rage & Escuillié, 2000; Caldwell & Lee, 1997; Tchernov *et al*., 2000; Coates & Ruta, 2000). Although these fossils were originally interpreted as retaining the ancestral limbed condition, a subsequent evaluation of their highly derived cranial morphology has led to the alternative hypothesis that these forms may instead represent cases of limb reacquisition from limbless ancestors (Fig. 2; Rieppel, Head & Meyer, 2003). The genetic basis of limb loss in snakes has been linked to mutations in the ZRS enhancer of the Sonic hedgehog (*Shh*) gene, a key regulator of limb outgrowth. In snakes, deletions within the ZRS reduce its responsiveness to transcription factors such as HOXD13 and ETS1, resulting in markedly diminished *Shh* expression and premature arrest of limb development (Leal & Cohn, 2016). However, in relatively basal extant lineages such as in pythons, some ZRS functionality is retained, sufficient to transiently activate *Shh*, initiating the hedgehog signalling pathway and the formation of proximal skeletal elements of the limb (Leal & Cohn, 2016; Kvon *et al*., 2016). Crucially, the GRN and developmental mechanisms involved in limb formation have been broadly preserved in snake genomes, likely due to their pleiotropic roles in the development of other structures, particularly the genitalia (Leal & Cohn, 2016). For example, *Tbx4*, a gene essential for hindlimb formation, is expressed in both the hindlimbs and developing hemiphalluses of iguana *Anolis sagrei* embryos, and similarly in the rudimentary hindlimbs and hemiphalluses of *Python regius* embryos. In snakes, the hindlimb enhancer B (HLEB) of the *Tbx4* gene has lost its role in hindlimb development, while retaining its function in genital development (Infante *et al*., 2015). Similar pleiotropic constraints likely preserved other components of the limb gene regulatory network, including *Hoxd13* enhancers, which remain functional in both the limb buds and developing hemipenes of pythons (Leal & Cohn, 2015, 2016), as well as other genes that are expressed in both hindlimbs and genitalia (*Fgf8*, *Shh*, *Wnt5a*, *Bmp4*, *Bmp7*; Yamada *et al*., 2006). Intriguingly, genitalia in both vertebrates and arthropods can be experimentally transformed into legs (Estrada & Sánchez‐Herrero, 2001; Stansbury & Moczek, 2014; Lozovska, *et al*., 2024), suggesting an underlying ancient homology between legs and genitalia. Altogether, this evidence suggests that apparent limb reversals in fossil snakes may represent the reactivation of a developmental program that was never entirely dismantled, requiring only minor regulatory changes to restore limb formation.

Homologous structures may be present throughout a lineage but exist in subtle or highly modified states that make their homology difficult to recognise based on morphology alone. This phenomenon has been termed ‘cryptic persistence of unrecognised homologs’ (Bruce & Patel, 2022, 2023). For example, many arthropod ectodermal outgrowths, such as insect wings (Bruce & Patel, 2020; Clark‐Hachtel & Tomoyasu, 2020), the carapace of the crustacean *Daphnia* (Bruce & Patel, 2022; Shiga *et al*., 2017), beetle abdominal teeth (Bruce & Patel, 2023; Linz, Hu & Moczek, 2019; Ohde *et al*., 2013), and various body wall plates found throughout arthropods (Bruce & Patel, 2020, 2022), share the same GRN, positional context on the body, and developmental mechanisms. However, the sheer diversity of these structures in size, shape, and function obscured an appreciation of their homology until comparisons could be made of their embryonic development and GRNs, which revealed their common developmental and evolutionary origin. Thus, trait reversals *via* pleiotropy, such as the aforementioned wing reversal in stick insects (Forni *et al*., 2022; Fig. 1B), can also occur through the cryptic persistence of unrecognised homologs.

Furthermore, it has been shown recently that some evolutionary changes to GRNs can become ‘interlocked’. This means that when a GRN is repurposed for a new function, the changes it undergoes can persist in the GRN in all the contexts where it is expressed, not just in the context it was originally modified for (Molina‐Gil *et al*., 2023). This suggests that GRN modifications linked to a lost trait could persist even after the trait itself is lost phenotypically. Eventually, a single mutational event – for example, a transposable element disrupting a cis‐regulatory element or the up‐regulation of a gene (Orteu *et al*., 2024; Cooper & Milinkovitch, 2025) – could then restore the precise spatial and temporal activity of the lost‐trait GRN and reinstate its expression. These are situations in which a mechanism for the reactivation of a GRN for a lost function can be envisioned, but we do not yet have a general understanding of this evolutionary transition (see Section VIII).

## V. REVERSAL BY PLASTICITY

Phenotypic plasticity is the possibility for an individual organism to express different phenotypes in response to different environmental conditions (Fusco & Minelli, 2010). In some extreme forms, this phenomenon is responsible for the presence or absence of very complex traits, such as head and prothoracic horns in many species of scarab beetles, in response to larval nutritional conditions (Moczek, 2010). A trait only expressed in response to extremely rare environmental cues could appear as lost in a lineage. However, simulations have demonstrated that the genetic machinery responsible for the plastic expression of a trait, along with its underlying GRN, can remain fully functional in the genome over long periods (Visser *et al*., 2021).

**Fig. 3:**
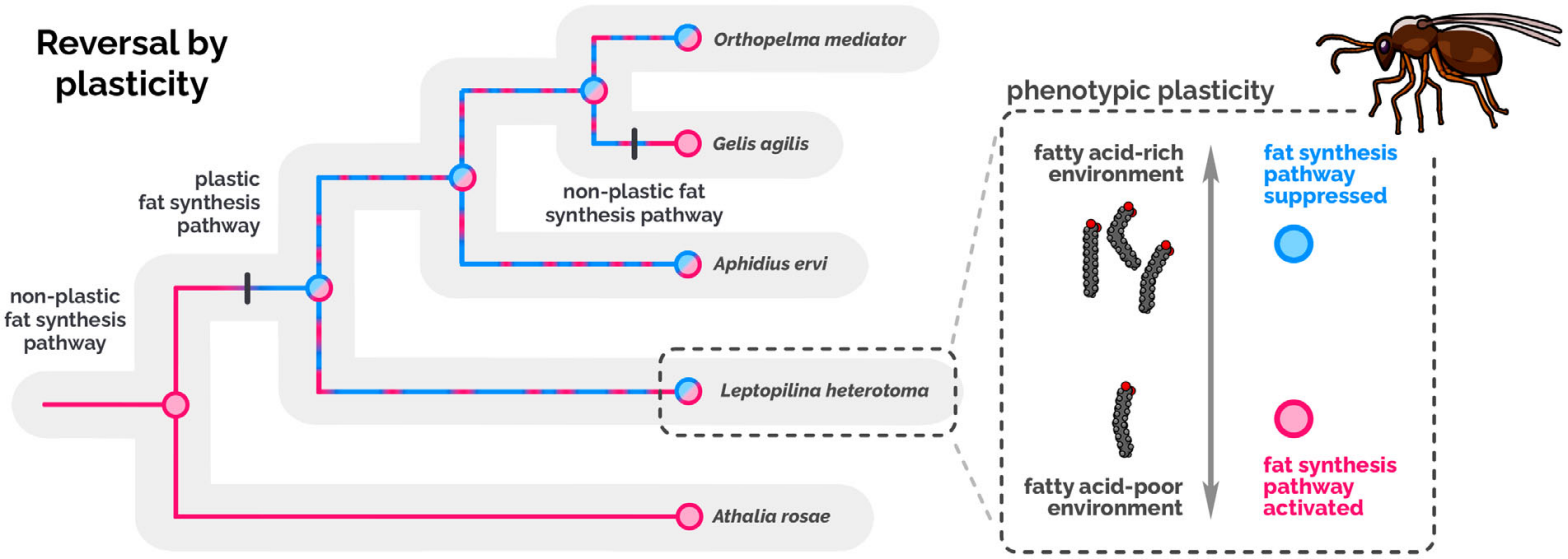
Reversal by plasticity. Phenotypic plasticity is the ability of an organism to produce different phenotypes with the same genomic background, in response to changes in environmental factors. Here, the absence of fat synthesis in the species of gall‐inducing wasp *Leptopilina heterotoma* (Cynipidae) has been shown to be due to phenotypic plasticity rather than a complete loss. When individuals are exposed to a fatty‐acid‐poor environment, the fat synthesis pathway is switched on, while the opposite happens in fatty acid‐rich environments. This may explain the reacquisition of constitutive fat synthesis in other parasitic wasps such as *Gelis agilis*. Although fat‐synthesis plasticity has been claimed for several parasitoid species, formal testing is still lacking in most cases. The cladogram topology is adapted from Visser *et al*. (2010, 2021). Pink = nodes and branches exhibiting the ancestral state of the character; alternating pink and blue = nodes and branches exhibiting the derived state of the character (plasticity); dashes highlight non‐plastic character expression loss and reinstatement.

A fascinating illustration of how plasticity can explain the reinstatement of a lost trait can be found in the proposed reinstatement of lipid synthesis in parasitoid wasps (Fig. 3). Most of the species of this group lack the ability to synthesise lipids, a trait supposedly lost more than 200 million years ago in the group's last common ancestor, in association with the adoption of a parasitic lifestyle and the consequent availability of lipids from the host. Visser *et al*. (2010) proposed that the fat synthesis pathway reverted independently many times in generalist species, following the variations in lipid availability across a wide range of hosts. However, a recent study on four parasitoid wasp species of the genus *Nasonia*, including the model species *N. vitripennis*, has shown that, contrary to the earlier assumptions, these wasps are able to synthesise lipids from sugars. Specifically, they can convert α‐D‐glucose into fatty acids (Prager, Bruckmann & Ruther, 2019), challenging previous findings that suggested a loss of this metabolic ability (Visser *et al*., 2012; Lammers *et al*., 2019). Other studies have revealed that some populations of the parasitoid wasp *Leptopilina heterotoma* are also capable of synthesising lipids. Furthermore, analyses based on both mitochondrial and nuclear genetic markers indicate that these lipid‐synthesising populations do not represent distinct species from those that appear unable to synthesise lipids (Visser *et al*., 2018). Instead, *L. heterotoma* can switch lipogenesis on in fat‐poor hosts and off in fat‐rich ones (Visser *et al*., 2021). Altogether, evidence suggests that the metabolic pathway for lipid synthesis was not completely lost in the ancestor of parasitoid wasps but persisted through phenotypic plasticity and was eventually constitutively reinstated in some species (Visser *et al*., 2021).

Additional examples of reversal by plasticity include instances where males are produced only under exceptional conditions, such as in a recently described species of darwinulid crustacean, *Vestenula cornelia* (Ostracoda). Darwinulids were believed to have been exclusively parthenogenetic for approximately 200 million years. However, the production of males in *V. cornelia* may represent a response to certain environmental or seasonal factors, similar to other non‐exclusively parthenogenetic taxa, such as most water fleas (Smith, Kamiya & Horne, 2006). Phenotypic plasticity has also been proposed to explain the maintenance of fully developed eyes and other features commonly associated with surface life, like pigmentation, in cavefish. For instance, the cyprinid cave fish *Telestes karsticus*, when reared in light conditions, can develop phenotypic features, including eye size, comparable to those of surface fish (Čupić *et al*., 2023).

## VI. REVERSAL BY HEMIPLASY

Hemiplasy is defined as a topological discordance between a gene tree and the species tree that comprises it (Avise & Robinson, 2008). Individual genes or loci can have different phylogenetic relationships from species' phylogenetic relationships (Maddison, 1997), mainly due to (*i*) incomplete lineage sorting [i.e. the retention of ancestral polymorphisms through multiple, successive speciation events (Pamilo & Nei, 1988; Doyle, 1997)] and (*ii*) reticulation, including introgression (i.e. the gene flow from the hybrid offspring to the parental generation following backcrossing; Rosser *et al*., 2024) and hybrid speciation (i.e. the generation of a new species by interbreeding between distinct lineages). In general, the signatures of these events in the genome tend to persist for a relatively long time (Suh, 2016; Scornavacca & Galtier, 2017; Cai *et al*., 2021). As such, hemiplasy presents itself as a candidate mechanism of reversal, involved in both ancient and recent events (Guerrero & Hahn, 2018), through a form of persistence occurring in a lineage different from that in which the reversal takes place. In other words, the reinstatement of a trait in a lineage that lost it could be obtained through merging with a closely related lineage where the same trait was retained. This kind of character reversal is more strictly associated with forms of asymmetric reticulation, such as introgression, where the resulting lineage retains continuity with that of the parental species – typically the one that had lost the character – and thus genuinely exhibiting a trait reversal. By contrast, symmetric reticulation, as observed in hybrid speciation, produces a novel taxonomic entity with mixed ancestry, so that the event less neatly qualifies as a true reversal.

**Fig. 4:**
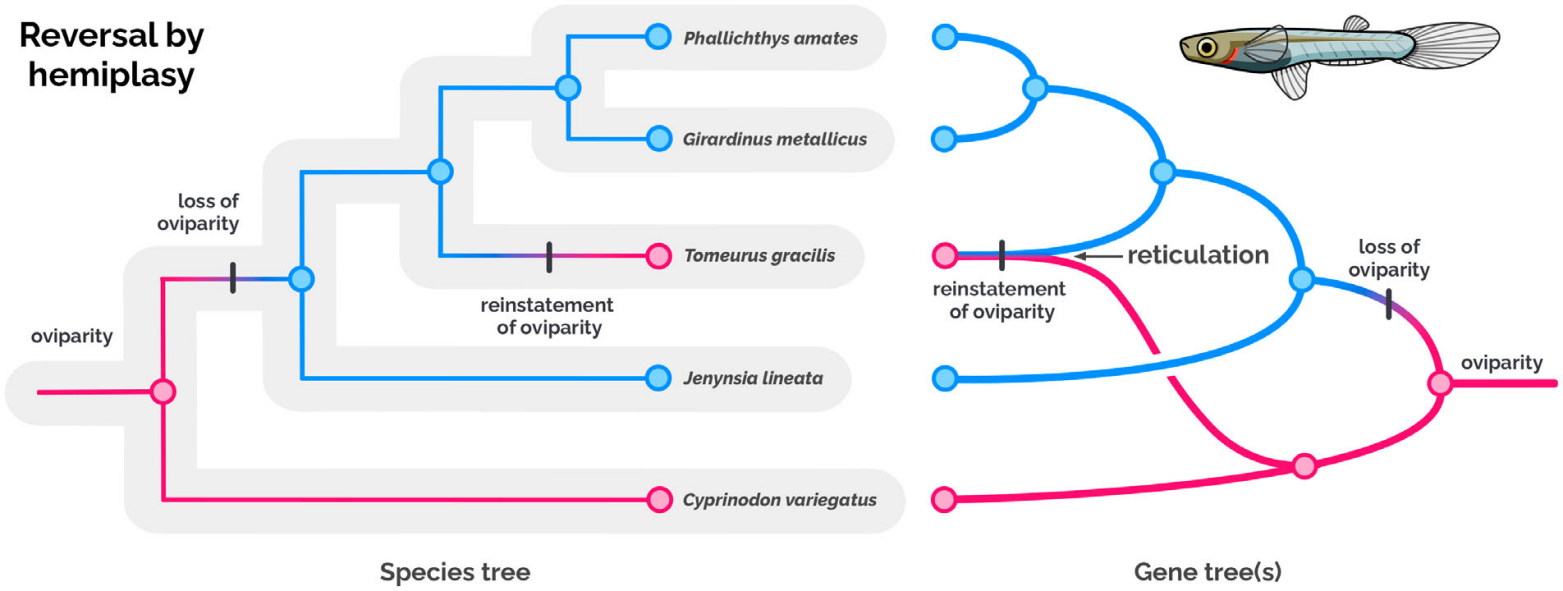
Reversal by hemiplasy. Hemiplasy is a phenomenon that can result in the reinstatement of a lost trait as a consequence of gene transmission not strictly following species tree topology. In the cyprinodontiform *Tomeurus gracilis*, the reacquisition of oviparity from a viviparous reproductive mode, as suggested by the species tree (left panel), can be explained by reticulate evolution (in this case introgression) between a viviparous and an oviparous species (right panel). The cladogram topology is adapted from Horreo *et al*. (2020). Pink = nodes and branches exhibiting the ancestral state of the character; blue = nodes and branches exhibiting the derived state of the character; dashes highlight loss and reinstatement.

Despite phylogenomic studies revealing that discordance between gene trees and species trees is rampant across the Tree of Life (Hime *et al*., 2021; Morales‐Briones *et al*., 2021), hemiplasy is rarely considered in explaining trait reversal (Hibbins, Gibson & Hahn, 2020; Wang *et al*., 2021), and clear cases attributable to this phenomenon are scarce. One is that of the poecilid fish *Tomeurus gracilis* (Cyprinodontiformes), where species‐tree analyses suggest it shifted from live bearing (viviparity) back to ancestral egg laying (oviparity) (Reznick *et al*., 2017). Here, phylogenetic‐network analyses uncover reticulation across an oviparous cyprinodontid ancestor – likely close to *Cyprinodon variegatus* – and the ancestor of *T. gracilis*, suggesting introgression as the most parsimonious explanation for the species' apparent reversal to the once‐lost oviparity (Fig. 4; Hórreo, Suárez & Fitze, 2020). At a shallower taxonomic level, the potentials of this mode of reversal have been experimentally tested by the partial reversal of visual function through artificial hybridisation among independently evolved populations of the blind cavefish *Astyanax mexicanus* (Wilkens & Strecker, 2003; Borowsky, 2008) and between cave and surface populations of the European freshwater fish *Barbatula barbatula* (Behrmann‐Godel *et al*., 2024).

Cases of trait reversal through reticulation are expected to be even more frequent in microbes, where horizontal gene transfer is a widespread process. For example, it has been shown how the independent loss of the GAL pathway gene cluster in yeasts was repeatedly reverted through horizontal gene transfer (HGT) tens of millions of years after their initial losses (Haase *et al*., 2021). Similarly, the genes required to produce the light‐harvesting pigment phycocyanin were lost in the ancestor of the cyanobacterium *Acaryochloris marina*, but then subsequently regained *via* HGT in a particular strain (Ulrich & Miller, 2024).

Reversal through hemiplasic persistence in evolutionary patterns may also occur through the acquisition of non‐homologous exogenous genes, rather than by reactivating the ancestral developmental pathway based on homologous GRNs. The recent acquisition of bacterial genes through HGT appears to have facilitated the transition from a derived parasitic lifestyle to an ancestral free‐living one in the protist genus *Trepomonas* (Xu *et al*., 2016), and the reinstatement of the alcoholic fermentation pathway in a clade of yeast living in floral nectar (*Wickerhamiella/Starmerella* clade; Gonçalves *et al*., 2018).

## VII. OVERLAPPING MECHANISMS IN TRAIT PERSISTENCE AND REVERSAL

The three modes of reversal characterised here are not mutually exclusive, as exemplified by the reinstatement of amphigonic reproduction (biparental sexual reproduction) from ancestors reproducing by parthenogenesis (uniparental reproduction through eggs that do not need to be fertilised) (Fusco & Minelli, 2019). This macroevolutionary pattern has been proposed, for instance, in reptiles of the families Alopoglossidae, Cordylidae, and Gerrhosauridae (Squamata; Moreira, Fonseca & Rokas, 2021) and in Crotoniidae oribatid mites (Acari; Domes *et al*., 2007). In the reversal to amphigony, pleiotropy, plasticity, and hemiplasy could be at play. Physiological pathways associated with male gonads have been proposed to have pleiotropic effects on female gonads, preventing the underlying genes from decaying in the absence of males (van der Kooi & Schwander, 2014; Forni *et al*., 2024). Likewise, recent studies provide an increasing number of cases of cryptic sex and sporadic emergence of males in previously believed obligate parthenogens (Vakhrusheva *et al*., 2020; Boyer *et al*., 2021; Scali, 2013; Freitas *et al*., 2023), suggesting a role for plasticity in the reinstatement of amphigonic reproduction. Lastly, it has been proposed that gene flow between amphigonic and parthenogenetic lineages can result in hemiplasy and eventually contribute to the reinstatement of amphigonic reproduction in both *Timema* stick insects (Larose *et al*., 2023) and *Darevskia* lizards (Danielyan, Arakelyan & Stephanyan, 2008; Carretero *et al*., 2018).

## VIII. A LOOK BEYOND THE CONTRAST BETWEEN OLD AND NEW CHARACTERS

In apparent contrast with Dollo's law, the reinstatement of lost traits is a recurrent macroevolutionary pattern throughout the Tree of Life. In the present work, we highlighted how the generative processes of the phenotypically lost trait could have survived the loss. This may have occurred because of the involvement of the same generative components in other traits and/or functions (pleiotropy); by environment‐dependent expression of the trait (plasticity); through extra‐lineage endurance followed by reticulation (hemiplasy); or by a combination of the three. The persistence of a GRN, beyond the decay and/or loss of the trait it contributes to, has wide experimental support. These findings echo recent discoveries about another remarkable macroevolutionary feature: the emergence of so‐called *evolutionary novelties*.

While there are multiple definitions of evolutionary novelty (e.g. focusing on functional or structural rationales; Minelli & Fusco, 2005), these are generally conceptualised as features that have no obvious homology with other features of the same organism, and whose origin cannot easily be traced back to a modification of a feature existing in an ancestral species (Müller & Wagner, 1991; Minelli & Fusco, 2013). The origin of novel traits has always been a topic of special interest in the study of evolutionary history. However, similarly to what is observed in the cases of reversal discussed so far, in several instances, ‘novel’ phenotypes and functions appear to have originated from modifications of pre‐existing gene networks or morphogenetic fields, as in the case of beetle head horns (Hu, Linz & Moczek, 2019; Linz & Moczek, 2020), the water flea carapace (Shiga *et al*., 2017; Bruce & Patel, 2020, 2022), mammalian flight membranes (Feigin *et al*., 2023), and snake venom (Barua & Mikheyev, 2021). Thus, while novelties and reversals can be clearly distinguished as macroevolutionary patterns (schematically, as character state transition A–B *versus* A–B–A, respectively), the commonalities of their underlying mechanisms point to a blurred demarcation in evolution between ‘old’ and ‘new’, or between ‘reinstated’ and ‘never really lost’. As a possible distinction, a reinstated trait would be expected to resemble more the ancestral trait in both form and function, with the terminal components of the GRN more similar to that of the ancestral trait, whereas a novel trait would be expected to look and function somewhat differently from the ancestral trait, with terminal components of the GRN less similar to that of the ancestral trait. However, this also implies that the difference between reinstated and novel traits falls along a continuum.

This broader view on the relationship between primitively lost and newly reinstated traits provides a more comprehensive framework for interpreting evolutionary reversals. Morphological and developmental differences between ancestral and reinstated traits (Wagner *et al*., 2018; Yang *et al*., 2023; Forni *et al*., 2023) suggest that, beyond the simplicity of the schematic pattern of character state transition identifying the reversal (A–B–A), this is actually a complex evolutionary change, and that understanding these transitions requires an assessment of the developmental and evolutionary mechanisms underlying the reinstatement. A first, basic assessment is whether the trait once lost in an ancestor was reinstated in a descendant through a completely different developmental pathway, controlled by a different set of identity‐conferring genes in a different tissue and a different positional context, thus producing a genuinely homoplastic re‐evolved trait; or conversely, if the reinstatement uses the same developmental pathway, controlled by the same identity‐conferring genes, in the same tissue, and in the same positional context thus giving rise to a re‐evolved trait that can be placed in homology relationship with an ancestral trait. The former case can be exemplified by the occurrences of reversal realised through the acquisition of (exogenous) bacterial genes through HGT in some unicellular eukaryotes, protists, and yeasts mentioned above. Remarkably, there are apparently no documented cases where the reinstatement of a trait was attained through the co‐option of genes or GRNs involved in the development of other traits in the same species. This pattern may partly reflect the relatively limited functional genomics resources available for non‐model species (Elmer & Clobert, 2024).

In the latter case, when the reversal is founded on the persistence of the developmental machinery underlying the lost trait – whether *via* pleiotropy, plasticity or hemiplasy – the reinstated trait is expected to exhibit different elements of morphological and molecular homology to the ancestral trait. For example, with respect to reversals by pleiotropy, this may depend on the degree of overlap between the terminal GRNs of the lost feature and that of other feature(s) which have maintained historical continuity along the lineage. Conversely, in instances of reversals by plasticity and hemiplasy, homology with the ancestral trait is expected to be more pervasive, since the maintenance of the GRN is related to a form of conservation of the trait itself.

Nonetheless, a key knowledge gap persists: we still lack a detailed understanding of how these retained developmental elements can be reactivated. In some cases, a single mutational event, such as a transposable element disrupting a repressor site or the upregulation of a key regulatory gene (Orteu *et al*., 2024; Cooper & Milinkovitch, 2025), may suffice to reinitiate the spatiotemporal activity of the GRN associated with the lost trait. Additionally, because the phenotypic loss of a trait often occurs by degenerating its embryonic precursor after this has already formed, reinstatement may involve preventing this degeneration, for instance by blocking apoptosis or maintaining survival signals. However, beyond what is suggested by a few specific studies, it is not known if there is any general pathway for the reactivation of a silent GRN or for its re‐involvement in a lost function.

## IX. CONCLUSIONS

(1) Current evidence indicates that trait reversal is more widespread than traditionally assumed, challenging the long‐standing view that trait loss is irreversible. However, although instances of such a process are increasingly documented across diverse taxa and traits, a comprehensive process‐based conceptual framework is still lacking.
(2) Here we identify three primary evolutionary phenomena – pleiotropy, plasticity, and hemiplasy – that, individually or in combination, may enable the persistence of the molecular and developmental elements underlying lost traits. These processes can create the potential for trait reinstatement over large evolutionary timescales.
(3) Although numerous studies suggest the contribution of these three phenomena, with varying lines of evidence depending on the case, a critical gap remains as we still lack a mechanistic understanding of how preserved developmental resources can be involved in trait reversal.
(4) Trait reversal and evolutionary novelty, despite appearing to embody opposite patterns of evolutionary change, may in fact occur *via* the same underlying mechanisms of repurposing latent developmental resources: the reactivation of a conserved GRN (reversal), or the modification of a GRN to make a new feature (novelty). Acknowledging this shared potential mechanism undermines any rigid divides between ‘old’ and ‘new’ in evolution.
(5) The exploration of the developmental mechanisms behind the macroevolutionary phenomenon of trait reversal has just started and it will eventually shed light on the way(s) evolution can retrace its steps.
